# Herpes Zoster virus infection and the risk of developing dementia: A systematic review and meta-analysis

**DOI:** 10.1097/MD.0000000000034503

**Published:** 2023-10-27

**Authors:** Rowan H. Elhalag, Karam R. Motawea, Nesreen Elsayed Talat, Samah S. Rouzan, Sarraa M. Reyad, Soliman M. Elsayed, Pensée Chébl, Marwan Abowafia, Jaffer Shah

**Affiliations:** a Faculty of Medicine, Alexandria University, Alexandria, Egypt; b Weill Cornell Medicine, New York, NY, USA.

**Keywords:** AD, Dementia, HZV, meta-analysis

## Abstract

**Background:**

Herpes Zoster, commonly known as shingles, is a viral infection that affects a significant portion of the adult population; however, its potential role in the onset or progression of neurodegenerative disorders like dementia remains unclear.

**Methods:**

We searched the following databases: PubMed, Scopus, Cochrane library, and Web of Science. We included any randomized control trials and controlled observational studies as Cross-sectional, prospective, or retrospective cohort and case-control studies that investigated the prevalence of dementia in Herpes Zoster Virus (HZV)-infected patients and HZV-free control group or if the study investigated the prevalence of HZV in demented patients. Also, if the studies measured the levels of dementia biomarkers in patients with HZV compared with a healthy control group.

**Results:**

After the complete screening, 9 studies were included in the meta-analysis. In the outcome of the incidence of HZV, the pooled analysis showed no statistically significant difference between the dementia group and the No dementia group (RR = 1.04% CI = 0.86–1.25, *P* = .70). In the outcome of incidences of dementia and Alzheimer’s disease, the pooled analysis showed no statistically significant difference between the HZV group and the incidence of dementia (RR = 0.99, 95% CI = 0.92–1.08, *P* = .89), (RR = 3.74, 95% CI = 0.22–62.70, *P* = .36) respectively. In the outcome of incidences of Herpes Zoster ophthalmicus (HZO), the generic inverse variance showed a statistically significant association between patients who have HZO and increased incidence of dementia (RR = 6.26, 95% CI = 1.30–30.19, *P* = .02).

**Conclusion:**

Our study showed no significant association between HZV and the incidence of dementia or Alzheimer’s disease, but it shows a significant association between HZO and the incidence of dementia. More multicenter studies are needed to establish the actual association between the HZV and dementia.

## 1. Introduction

Dementias are the primary global cause of dependency and disability among older people, and they also contribute significantly to disease burden in established market economies, according to data from the World Health Organization.^[1]^ At the moment, 5% of those over 65 have dementia, and that percentage rises to about 50% for people over 90.^[2]^ Age is the largest risk factor for dementia because it occurs in more than 90% of cases after the age of 65. As the average population age increases, dementia incidence and prevalence are increasing globally.^[3]^ There are currently 46.8 million dementia sufferers globally, and by the year 2050, that number is expected to rise to 131.5 million, according to the World Alzheimer Report, a detailed meta-analysis of population-based studies.^[4]^ The most common dementia subtypes include Alzheimer’s disease (AD), vascular dementia, frontotemporal dementia and related syndromes, Lewy body dementias, and prion disorders.^[3]^ AD, by far the most common dementia etiology, accounts for up to 80% of dementia diagnoses.^[5]^ Mortality attributable to AD increased by 89% between 2000 and 2014.^[6]^

The past decade has experienced tremendous growth in the degree of investigation for AD biomarkers. The amyloid cascade hypothesis is the dominant theory that aims to explain the onset and pathogenesis of AD mechanistically.^[7]^ It was first postulated in the late twentieth century, following the revelation that chromosome 21 mutations involved defective amyloid precursor protein (APP) metabolism which resulted in hazardous Aβ peptide deposition. In AD, some of the broken protein fragments combine into amyloid, which then accumulates.^[8]^ These hazardous protein aggregates have been shown to alter synapse function, and brain development, limit long-term potentiation (LTP), induce neurodegeneration, and also cause gliosis.^[9–11]^ Since Schenk et al’s^[12]^ study, numerous research targeting these beta-amyloid proteins have been done. In a recent clinical trial, van Dyck et al,^[13]^ showed that lecanemab reduced amyloid indicators in early AD and resulted in a moderately slower loss in cognition and function assessments than placebo after 18 months, however, it was related to adverse effects.^[13]^ Faced with data that calls the Amyloid hypothesis into question, researchers have reconsidered the role of amylin, also known as islet amyloid polypeptide, in the pathophysiology of AD.^[14]^ Human amylin was discovered in the pancreas, where it is released alongside insulin by pancreatic B cells.^[15]^ According to endocrinology studies, Amylin levels are higher than usual in the early stages of Type II diabetes mellitus. Furthermore, in the absence of a corresponding amount of stabilizing insulin, this protein is prone to misfolding and the formation of oligomers and fibrils.^[15,16]^ Taking things a step further, research has revealed that type 2 diabetes mellitus is a substantial risk factor for the development of AD.^[17]^ Research has shown that these peptides cause the death of neurons via the induction of proapoptotic genes in a mechanistically similar way to Aβ plaques.^[18]^ These data suggest that amylin, like Aβ peptides, is cytotoxic to neurons and pathogenic, leading to AD.

Varicella-zoster virus (VZV) is a neurotropic human herpes virus in the alpha herpesviridiae genus. It is a virus that causes both chickenpox/varicella and shingles/Herpes zoster (HZ). The virus is responsible for primary infection which results in varicella while HZ reflects the reactivation of latent infection.^[19]^ The virus’s reactivation may encourage neuroinflammation by causing the formation of misfolded oligomers, the buildup of amyloid plaques, and the development of neurofibrillary tangles made of hyper-phosphorylated tau protein.^[20–22]^ VZV may also directly infect astrocytes to encourage intracellular amyloid formation and extracellular amyloid fibril aggregation.^[23]^ HZ occurs everywhere around the world with no seasonal fluctuations. The prevalence of HZ varies by age, from 1.2 to 3.4 per 1000 individuals per year in younger folks to 3.9 to 11.8 per 1000 individuals per year in patients over the age of 65.^[24]^

VZV involvement of the trigeminal nerve’s ocular division (V1) is known as HZO. Ocular symptoms such as conjunctivitis, uveitis, episcleritis, keratitis, or retinitis affect 50% to 85% of HZO cases.^[25,26]^ Due to the possibility of visual loss, HZO is considered an ophthalmologic emergency.^[19]^ VZV is the only human virus that is capable of replication in cerebral arteries and causes vasculopathy. It primarily affects the elderly as well as those with impaired immune systems.^[27,28]^ Additionally, via the trigeminal nerve, namely from the ophthalmic branch of trigeminal afferent fibers, the virus travels trans-axonally to cerebral arteries causing additional vascular inflammation and thrombosis that may subsequently damage brain cells.^[29–31]^ In light of these findings, research by Bennett et al concluded that even in younger patients, those with HZO have a 1.3- to 4-fold higher risk of cerebrovascular events after the disease.^[32]^ Moreover, multiple studies revealed that having a stroke contributes to raising the possibility of dementia.^[33–35]^ Thus, HZO may be regarded as a common risk factor in the development of dementia and VZV vasculopathy.^[36]^

Finally, on searching the literature we found that several studies reported a possible association between HZV infection and an increased risk of developing dementia.^[37–39]^ On the other hand, 2 studies have contradictorily reported no significant association between HZV infection and increased risk of dementia.^[40,41]^ Thus, we conducted this meta-analysis to resolve the controversy and evaluate whether HZV infection increases the risk of dementia or not.

## 2. Methods

This systematic review and meta-analysis were performed according to the Preferred Reporting Items for Systematic Reviews and Meta-Analyses (PRISMA) guidelines.

### 2.1. Search strategy

A literature search of the following databases (PubMed, Scopus, and Web of Science) on the 11th of September 2022, using key terms such as (Herpes zoster OR shingles OR Chickenpox) AND (Dementia OR Alzheimer OR mild cognitive impairment) was performed to identify relevant studies.

### 2.2. Inclusion and exclusion criteria

We screened studies by titles and abstracts according to the following criteria: Any randomized control trials and controlled observational studies as Cross-sectional, prospective, or retrospective cohort and case-control studies that investigated the prevalence of dementia in HZV-infected patients and HZV-free control group or if the study investigated the prevalence of HZV in demented patients. Also, if the studies measured the levels of dementia biomarkers in patients with HZV compared with a healthy control group. No age restriction was implicated.

#### 2.2.1. Exclusion criteria.

Non-controlled studies, case reports, case series, editorials, animal studies, and brain specimen-based studies of dead patients.

### 2.3. Study selection

Two independent reviewers screened the titles and abstracts of the studies according to our criteria. If an agreement is not achieved, a third opinion was obtained from the first author to resolve the conflict.

### 2.4. Data extraction and quality assessment

Some authors were assigned the studies to perform data extraction, where each study was extracted by 2 reviewers independently.

The following baseline data were extracted from the included studies: the first author of the study, year of publication, study design, arms of the study, number of participants, age of participants, sex of participants, medical condition, and other baseline medications. And the following outcomes were extracted:

Incidences of dementia, Incidences of Alzheimer’s disease (AD), Amylin, Aβ40, Aβ42, Amyloid, Incidence of Herpes Zoster Virus (HZV), and Herpes Zoster ophthalmicus (HZO).

The quality was assessed using Newcastle-Ottawa Scale, for evaluating the quality of observational studies. Each study was ranked as good, fair, or poor quality according to its score.

### 2.5. Data synthesis

Data were analyzed using RevMan software, version 5.4. Continuous data were presented as mean difference (MD) with a 95% confidence interval (CI). Dichotomous data were presented as risk ratio (RR) with a 95% CI. If no heterogeneity was observed, results were presented in a fixed effect model and a random effect model was used if significant heterogeneity was observed. Sensitivity analysis (leave-one-out test) will be used to resolve the heterogeneity if detected. Results were considered significant if the *P* value was less than .05.

## 3. Results

### 3.1. Summary of studies

**Figure 1.**
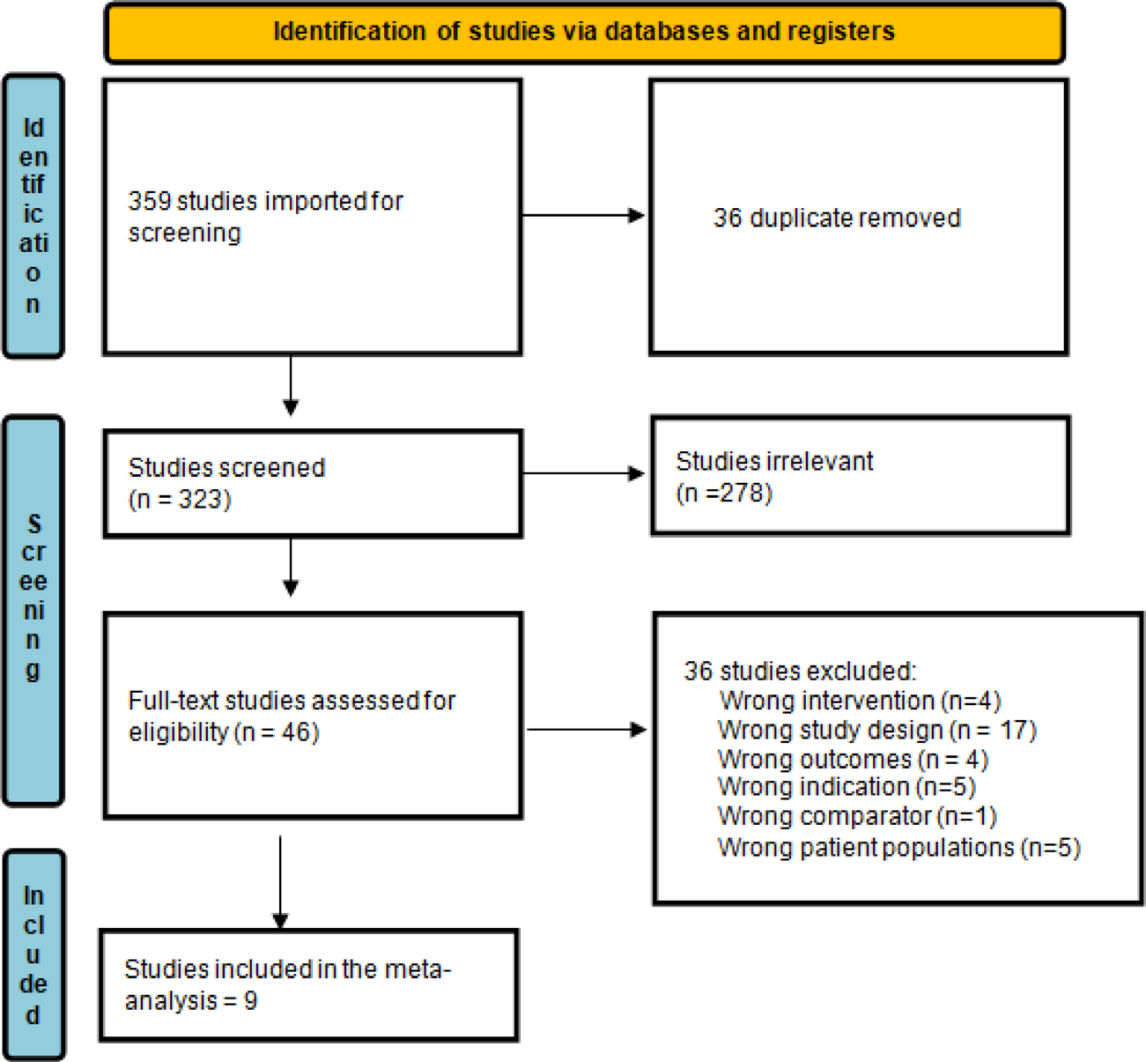
Prisma flow diagram.

After searching the literature, 359 studies resulted, and then became 323 were eligible for the title and abstract screening after duplicate removal. Of the 323, 278 were irrelevant as they didn’t meet the inclusion criteria of our study and finally 46 studies were eligible for full-text screening. Finally, 9 studies were included in the meta-analysis after the full-text screening, as shown in the PRISMA in (Fig. 1).

We have compared HZV and no HZV according to the outcomes: incidence of dementia, the incidence of AD, Amylin levels, Aβ40 levels, Aβ42 levels, and Amyloid levels in 3, 2, 2, 3, 3, 2 studies respectively. The incidence of HZV was compared between the dementia group and the no dementia group in 2 studies. The incidence of dementia in patients who have herpes zoster ophthalmicus (HZO) was pooled in 3 studies by generic inverse variance analysis.

The overall quality was good in most of the included studies, as shown in Table 1.

**Table 1:** Quality assessment.

NOS scale risk of bias assessment										
	Selection				Comparability	Exposure			Total score	AHRQ standards
Study	Case definition	Representativeness	Selection of controls	Definition of controls	Comparability	Ascertainment	Same method	Non-response rate	0	
1) Omland 2021	1	1	0	0	2	1	1	0	6	Good
2) Bubak 2020	1	0	1	1	2	1	1	1	8	Good
3) Bubak 2021	1	0	0	1	2	1	1	1	7	Good
4) Choi 2021	1	0	1	1	0	1	1	1	6	Fair
5) Shinomoto 2021	1	0	1	1	2	1	1	0	7	Good
6) Lophatananon 2020	1	0	1	1	2	1	1	0	7	Good
7) Warren-Gash 2022	1	0	1	1	2	1	1	1	8	Good
8) Tsai 2017	1	1	1	1	1	1	1	1	7	Good
9) Schmidt 2022	1	0	1	0	2	1	1	0	6	Fair

NOS = Newcastle-Ottawa Scale.

The total number of patients included in the study is 3090813 patients, 852,837 patients in the HZV group, 1,952,615 in the no HZV control group, also 13816 patients in the dementia group, and 271,545 in no dementia group, other baseline data are shown in Table 2.

**Table 2:** Baseline characteristics.

ID	Study design	Arms of the study	Number of patients in each group		Age (yr)		Sex (n)				Medical conditions		Baseline medications	
			Case	Control	Case	Control	HZV		No HZV					
							Female	Male	Female	Male	Case)	Control)	Case)	Control)
Schmidt 2022	Retrospective cohort	2 (HZ – control)	247,305	123,5890	median age (IQR) = 64 (54–74)	median age (IQR) = 64 (54–74)	149,796	97,509	748,427	487,464	No. of event (percentage from total) Chronic kidney disease 4368 (1.8) Chronic obstructive pulmonary disease 14,662 (5.9) Asthma 4069 (1.6) Hematologic cancer 4900 (2.0) Solid cancer 25,619 (10.4) Diabetes 19,485 (7.9) HIV 441 (0.2) Hyperlipidemia 56,375 (22.8) Traumatic head injury 7328 (3.0) Stroke 12,054 (4.9)	No. of event (percentage from total) Chronic kidney disease 11,993 (1.0) Chronic obstructive pulmonary disease 51,742 (4.2) Asthma 15,004 (1.2) Hematologic cancer 7580 (0.6) Solid cancer 100,827 (8.2) Diabetes 89,441 (7.2) HIV 504 (0.0) Hyperlipidemia 248,140 (20.1) Traumatic head injury 34,181 (2.8) Stroke 53,630 (4.3)	No. of event (percentage from total) Glucocorticoids 12,072 (4.9) lipid lowering therapy 56,375 (22.8)	No. of event (percentage from total) Glucocorticoids 24,581 (2.0) lipid lowering therapy 248,140 (20.1)
Warren-gash 2022	Retrospective cohort	2 (HZ – control)	177,144	706,901	Mean (SD) = 65.1 (12.9)	Mean (SD) = 65.1 (12.8)	106,454	70,690	424,840	282,061	No. of events (percentage of total) Obese 41,483 (23.4) Active Smoking 35,960 (20.3) Ex smoker 74,081 (41.8) Harmful alcohol use 6071 (3.4) Asthma 26,956 (15.2) COPD 19,931 (11.3) Diabetes 20,725 (11.7) Uncontrolled diabetes (2 years) 9271 (5.2) Hypertension (diagnosed) 62,436 (35.2) Ischaemic heart disease 34,429 (19.4) Stroke 6595 (3.7) Traumatic brain injury 1454 (0.8) Immunosuppressed 19,623 (11.1) Autoimmune disease 18,015 (10.2) Liver disease 1359 (0.8) Chronic kidney disease 15,181 (8.6) Depression (2 years) 4316 (2.4) HSV 8535 (4.8)	No. of event (percentage of total) Obese 157,956 (22.3) Active Smoking 155,237 (22.0) Ex smoker 267,329 (37.8) Harmful alcohol use 24,601 (3.5) Asthma 87,768 (12.4) COPD 63,536 (9.0) Diabetes 75,524 (10.7) Uncontrolled diabetes (2 years) 32,671 (4.6) Hypertension (diagnosed) 232,748 (32.9) Ischaemic heart disease 117,646 (16.6) Stroke 25,152 (3.6) Traumatic brain injury 5310 (0.8) Immunosuppressed 45,957 (6.5) Autoimmune disease 53,030 (7.5) Liver disease 4820 (0.7) Chronic kidney disease 51,882 (7.3) Depression (2 years) 14,310 (2.0) HSV 23,607 (3.3)	No. of event (percentage of total) Antivirals (in 7 days) 110,997 (62.7)	No. of event (percentage of total) Antivirals (in 7 days) 186 (<1)
Buback 2021	Case control study	2 (VZV vasculopathy subjects - stroke controls)	16	36	mean (SD) = 54.44 (15.76)	mean (SD) = 52.19 (16.13)	6	10	17	19	All patients have VZV vasculopathy	All controls have stroke		
Omland 2021	Retrospective cohort	2 (VZV antibodies in csf with HZ cases – control)	74	1406	median (IQR) = 65 (37–80)	median (IQR) = 65 (37–80)	42	32	798	608	No. of event (percentage of total) Comorbidity, n (%) = 45 (62) Immunosuppressive condition, n (%) = 20 (27) Transplantation (solid/hematopoietic), n (%) = 3 (4) HIV, n (%) = 2 (3) Cancer, n (%) = 12 (16) Inflammatory bowel disease, n (%) = 1 (1) Systemic lupus erythematosus, n (%) = 0 (0) COPD, n (%) = 3 (4) Rheumatoid arthritis, n (%) = 1 (1) Ankylosing spondylitis, n (%) =1 (1) Psoriasis, n (%) = 2 (3)	No. of event (percentage of total) Comorbidity, n (%) = 490 (35) Immunosuppressive condition, n (%) = 208 (15) Transplantation (solid/hematopoietic), n (%) = 4 (0) HIV, n (%) = 1 (0) Cancer, n (%) = 113 (8) Inflammatory bowel disease, n (%) = 19 (1) Systemic lupus erythematosus, n (%) = 2 (0) COPD, n (%) = 61 (4) Rheumatoid arthritis, n (%) = 16 (1) Ankylosing spondylitis, n (%) = 4 (0) Psoriasis, n (%) = 11 (1)		
Shinomto 2021	Case control study	2 (VZV with CNS involvement – control)	8	18	mean (SD) = 46.88 (26.36)	Mean (SD) = 51.56 (17.45)	4	4	8	10	No. of event Myelitis 1 Meningitis 6 Encephalitis 1	No. of event Influenza 1 Fever elevation 4 Crowned dens syndrome 1 Cervical spondylosis 2 Neuralgic amyotrophy 1 Fatigue 1 Oculomotor paresis 1 Dysphagia 1 Viral myositis 1 Transient global amnesia 1 Psychophysiologic disorder 1 Peroneal nerve paralysis 1 Brachial plexus neuropathy 1 Guillain-Barré syndrome 1	No. of events: Acyclovir: 8 (all cases) Corticosteroid: 4 (3 cases with encephalitis, 1 case of myelitis) Anti-epileptic medications: 2 (2 encephalitis cases) Vidarabine: 1 (1 case of encephalitis)	
Lophatananon 2021	Nested case-control	2 (dementia – control)	2378	225,845	mean age (SD) = 68.91 (6.51)	mean age (SD) = 65.35 (8.07)	1187	1191	123,685	102,160				
Choi 2021	Nested case-control	2 (dementia - control)	11445	45780	No. in each age group (percentage of total) 60–64 580 (5.1) 65–69 1289 (11.3) 70–74 2325 (20.3) 75–79 2979 (26.0) 80–84 2706 (23.6) 85 + 1566 (13.7)	No. in each age group (percentage of total) 60–64 2320 (5.1) 65–69 5156 (11.3) 70–74 9300 (20.3) 75–79 11,916 (26.0) 80–84 10,824 (23.6) 85 + 6264 (13.7)	7779	3666	31,116	14,664	No. of event (percentage of total) Hypertension = 8311 (72.6) Diabetes mellitus = 4065 (35.5) Dyslipidemia = 3552 (31.0) Ischemic heart disease = 1703 (14.9) Stroke = 5517 (48.2) Depression = 3237 (28.3) Herpes zoster infection = 928 (8.1)	No. of event (percentage of total) Hypertension = 33,244 (72.6) Diabetes mellitus = 16,260 (35.5) Dyslipidemia = 14,208 (31.0) Ischemic heart disease = 6004 (13.1) Stroke = 11,356 (24.8) Depression = 4637 (10.1) Herpes zoster infection = 3929 (8.6)		
Tsai 2017	Retrospective cohort	2 (Herpes zoster ophthalmicus – control)	846	2538	mean age (SD) = 62.2 (12.5)	mean age (SD) = 61.4 (13.3)	426	420	1220	1318	No. of event (percentage of total) Hypertension = 458 (54.1) Diabetes = 210 (24.8) Hyperlipidemia = 312 (36.9) Stroke = 162 (19.2) Coronary heart disease = 234 (27.7)	No. of event (percentage of total) Hypertension = 1321 (52.1) Diabetes = 569 (22.4) Hyperlipidemia = 910 (35.9) Stroke = 446 (17.6) Coronary heart disease = 657 (25.9)		
Buback 2020	Case control study	2 (HZ – healthy control)	14	10	mean (SEM) = 60.20 (3.86)	mean (SEM) = 50.00 (3.33)	7	7	5	5				

HZ = Herpes Zoster, VZV = varicella-zoster virus.

### 3.2. Outcomes

#### 3.2.1. Incidences of dementia.

**Figure 2.**
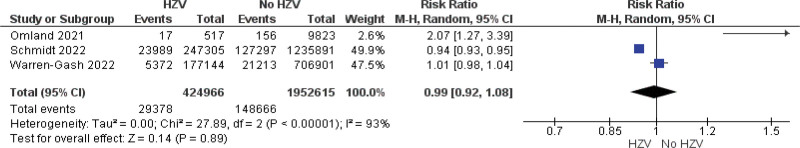
Forest plot explaining the incidences of dementia in the HZV group compared with the no HZV group. CI = confidence interval, HZV = Herpes Zoster Virus.

The pooled analysis showed no statistically significant difference between the HZV group and the incidence of dementia (RR = 0.99, 95% CI = 0.92–1.08, *P* = .89). We observed a significant heterogeneity among studies (*P* < .00001, *I*^2^ = 93%) that wasn’t solved by the leave-one-out test, Figure 2.

#### 3.2.2. Incidences of AD.

**Figure 3.**
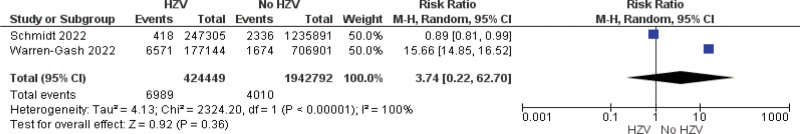
Forest plot explaining the incidences of AD in the HZV group compared with the no HZV group. AD = Alzheimer’s disease, CI = confidence interval, HZV = Herpes Zoster Virus.

The pooled analysis showed no statistically significant difference between the HZV group and the No HZV group (RR = 3.74, 95% CI = 0.22–62.70, *P* = .36). We observed a significant heterogeneity among studies (*P <* .00001, *I*^2^ = 100%), Figure 3.

#### 3.2.3. Amylin.

**Figure 4.**
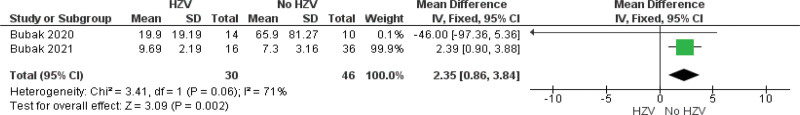
Forest plot explaining the presence of Amylin in the HZV group compared with the no HZV group. CI = confidence interval, HZV = Herpes Zoster Virus.

The pooled analysis showed a statistically significant association between the HZV group and an increased level of Amylin compared with the No HZV group (MD = 2.35, 95% CI = 0.86–3.84, *P* = 0.002). We observed no significant heterogeneity among studies (*P =* .06, *I*^2^ = 71%), Figure 4.

#### 3.2.4. Aβ40.

**Figure 5.**
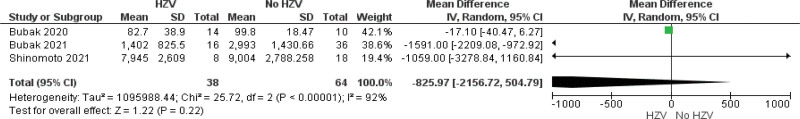
Forest plot explaining the presence of Aβ40 in the HZV group compared with the no HZV group. CI = confidence interval, HZV = Herpes Zoster Virus.

The pooled analysis showed no statistically significant difference between the HZV group and the No HZV group (MD = −825.97, 95% CI = −2156.72 to 504.79, *P* = .22). We observed a significant heterogeneity among studies (*P* < .00001, *I*^2^ = 92%) that was solved by leave-one-out test by removing Bubak 2020 (*P* = .65, *I*^2^ = 0%), and the analysis showed a statistically significant association between the HZV group and decreased levels of Aβ40 compared with No HZV group (MD = −1552.72, 95% CI = −2148.15 to −957.30, *P* < .00001), Figure 5.

#### 3.2.5. Aβ42.

**Figure 6.**
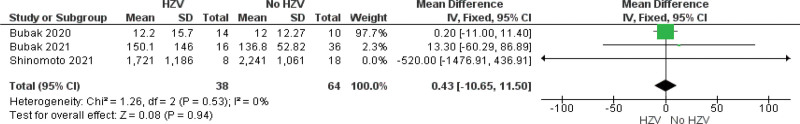
Forest plot explaining the presence of Aβ42 in the HZV group compared with the no HZV group. CI = confidence interval, HZV = Herpes Zoster Virus.

The pooled analysis showed no statistically significant difference between the HZV group and the No HZV group (MD = 0.43, 95% CI = −10.65 to 11.50, *P* = .94). We observed no heterogeneity among studies. (*P* = .53, *I*^2^ = 0%), Figure 6.

#### 3.2.6. Amyloid.

**Figure 7.**
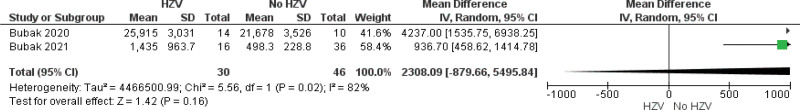
Forest plot explaining the presence of Amyloid in the HZV group compared with the no HZV group. CI = confidence interval, HZV = Herpes Zoster Virus.

The pooled analysis showed no statistically significant difference between the HZV group and the No HZV group (MD = 2308.09, 95% CI = −879.66 to 5495.84, *P* = 0.16). We observed a significant heterogeneity among studies (*P* = .02, *I*^2^ = 82%), Figure 7.

#### 3.2.7. Incidence of HZV.

**Figure 8.**
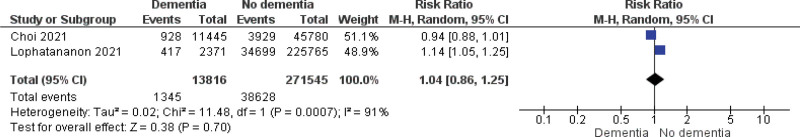
Forest plot explaining the incidence of HZV in the dementia group compared with the no dementia group. CI = confidence interval, HZV = Herpes Zoster Virus.

The pooled analysis showed no statistically significant difference between the dementia group and the No dementia group (RR = 1.04, 95% CI = 0.86–1.25, *P* = .70). We observed a significant heterogeneity among studies (*P* = .0007, *I*^2^ = 91%), Figure 8.

#### 3.2.8. HZO.

**Figure 9.**
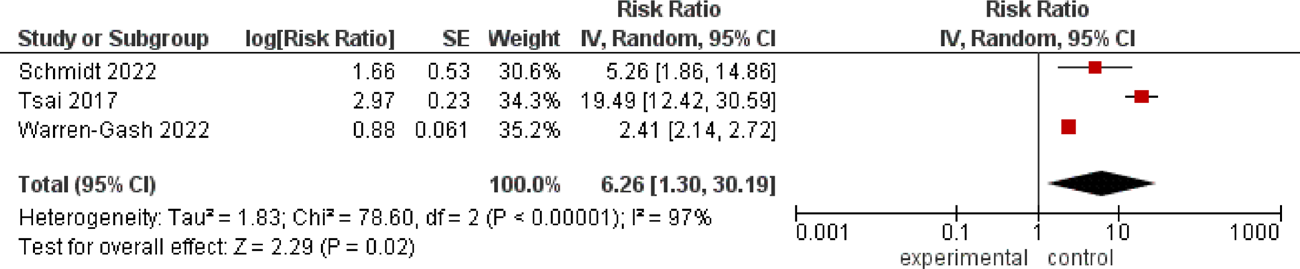
Forest plot explaining the association between patients who have HZO and the incidence of dementia. CI = confidence interval, HZO = Herpes Zoster Ophthalmicus.

The generic inverse variance showed a statistically significant association between patients who have HZO and an increased incidence of dementia (RR = 6.26, 95% CI = 1.30–30.19, *P* = .02). We observed a significant heterogeneity among studies (*P* < .00001, *I*^2^ = 97%), Figure 9.

## 4. Discussion

Our analysis of 9 studies with 3,090,813 patients revealed no statistically significant difference between the HZV group and no HZV group concerning the incidence of dementia, incidence of AD, and the increased level of Aβ40, Aβ42, and amyloid. However, we found a statistically significant association between the HZV group and an increased level of Amylin compared with the No HZV group. Also, no statistically significant difference was noticed when analyzing the incidence of HZV in the dementia group compared with the no HZV group. On the other hand, our study found a statistically significant association between patients who have HZO and an increased incidence of dementia.

Previous population-based studies reported both decreased and increased risks of dementia after HZ infection. However, our findings were in line with previous findings from other cohorts, such as Warren-Gash et al^[41]^ which, in a large UK population-based cohort, found no evidence that Herpes Zoster (HZ) was related to an elevated risk of a potential dementia diagnosis. Also on limiting exposure to HZO only, they found Comparable outcomes (adjusted HR 0.88, 95% CI = 0.78–0.99) for dementia. Furthermore, they identified a small apparent protective association between HZ (with or without antiviral use) and dementia which was confined to frail individuals and females and was seen only for mixed or unspecified dementia. Thus, using antiviral drugs did not affect their outcomes. Their findings were backed by a study from the SAIL Databank in wales.^[42]^ In the same study, individuals with treated HZ from Denmark had a 10% lower dementia diagnosis risk, while there was no difference in dementia diagnoses in Germany among those diagnosed and treated for HZ.^[42]^ These findings, however, conflict with 3 studies that used insurance claims data from Taiwan and Korea and found an increase in the risk of dementia following HZ,^[36,43]^ Nevertheless, only 2 studies^[43,44]^ found a significant decline in dementia among HZ patients after receiving antiviral treatment. However, they were unable to account for lifestyle factors and did not examine the influence of frailty. Additionally, the dementia outcome in the conflicting studies has varied. Either by requiring multiple instances of a dementia code in outpatient data^[43,44]^ or 30 days of medication.^[36]^ As a result, ascertainment bias may have occurred because dementia recording may have been more likely among those who sought care frequently. On the other hand, the sample size was unique to Warren et al.^[41]^ They included all patients with a record of HZ in a representative real-world UK population over 17 years, yielding a sample size at least twice as large as all prior research. In contrast to studies with conflicting findings, Warren-Gash et al^[41]^ managed to control for extra confounders. They carefully controlled for confounders and matched patients based on age, sex, practice, and calendar time; even though, they did not observe differences in the rates of dementia diagnoses between those exposed and unexposed to HZ. Finally, Warren-Gash et al^[41]^ also performed a variety of sensitivity assessments which in turn boosted the confidence in their estimations.

Another study that reinforced our belief in our estimates is Choi et al^[40]^ study. Data from the Korean National Health Insurance Service, which included information on over 1.14 million individuals, was used in this extensive nationwide cohort study in South Korea. By comparing 11,445 patients aged 60 or older with dementia to 45,780 matched controls without dementia, they discovered reduced odds of past HZ (OR 0.91; 95% CI = 0.84–0.97). In addition to basic characteristics like age, sex, income, and location of residence, the control group and the HZI group were matched for risk factors for dementia like hypertension, diabetes, and dyslipidemia. This careful matching offered reliable proof on the absence of a connection between dementia and HZI. Choi et al,^[40]^ have statistical power as they engaged large sample sizes. They employed a population-based data set made up of 1 million patients with a 12-year follow-up period to determine the relationship between HZI and dementia.

Taking into consideration that this study had limitations owing to the use of International classification of disease codes from administrative claims data and the number of visits patients made for dementia and HZI, which may not have been an accurate representation of the patient’s experience with dementia or HZI cases. Additionally, because doctors tend to take care of patients with HZV more frequently, they are more likely to receive outpatient or inpatient care, which increases the likelihood that they may be diagnosed with dementia. This observation can be the result of ascertainment bias.

Our meta-analysis reported a statistically significant association between patients who have HZO and an increased incidence of dementia. Thus, our study supports the idea that VZV reactivation rather than VZV primary infection is associated with dementia. Similarly, a study by Tsai et al^[36]^ included 2538 age-matched comparison participants and 846 patients with HZ ophthalmicus (HZO), with a mean age of 61.6 years. The comparison patients were chosen by matching them with a given patient with HZO in their consumption of medical services in the same index year. The patients were identified by first-time main diagnosis in clinics or hospitals. Within the 5 years following their index dates, the incidence rates of senile dementia were examined. This retrospective cohort study revealed that patients with HZO had a higher chance of developing dementia than patients without HZO, with 4.61% of patients with HZO receiving a dementia diagnosis over the 5-year follow-up period compared to 1.65% of patients without HZO (no details of any antiviral treatment were provided). In several population-based cohorts, dementia risk has also been attributed to herpes zoster ophthalmicus infections rather than other herpes zoster infections.^[36,40]^ It has been proposed that in cases of herpes zoster ophthalmicus infections, the virus is more likely to infiltrate the CNS compared to more peripherally located herpes zoster infections.^[45]^

Unfortunately, the previous research did not examine the relationship between neuroinflammation and dementia brought on by HZO. The high prevalence of dementia in HZO patients may be attributed to VZV vasculopathy, which may cause dementia by damaging cerebral neural cells as a result of cerebral artery inflammatory and thrombotic mechanisms.^[27–31,46,47]^ Prior research showed that patients with HZO were more likely to experience VZV vasculopathy and stroke than those with other variants of herpes zoster.^[46–48]^ Consequently, vascular dementia may be primarily linked to the risk of dementia brought on by HZI. Studies have also observed elevations in dementia following a stroke, ranging from 3% to 19% during different follow-up durations (from 3 months to 10 years).^[33–35]^ In addition, dementia and VZV reactivation may be influenced by aging, psychological stress, a lack of social support, and unfavorable life events.^[49–54]^ Thus, Tsai et al,^[36]^ tried to investigate the association between cerebrovascular events and increased risk of dementia in the HZO group. A history of stroke was taken into consideration in this study as a covariation factor, however, there was no difference in dementia incidence between the HZI and control groups. To investigate the association between HZO and dementia during the study period, a population-based dataset with a sizable sample size was assumed in this investigation. A significant statistical advantage was provided by the large sample size in terms of identifying actual differences between the 2 cohorts. Additionally, licensed neurologists, infection experts, and dermatologists with extremely good validity in Taiwan performed the HZO and dementia diagnoses. However, several limitations must be taken into consideration when interpreting the study’s findings. The biggest drawback is the lack of information regarding a person’s history of smoking, body mass index, education, or alcohol use, which are risk factors and may affect dementia pathophysiology.

In a population that was followed for up to 21 years, research by Schmidt et al found a minor relative risk of dementia in a HZ cohort (7%).^[55]^ Yet, the overall difference in absolute risk was negligible (less than 1%). It was surprising, nevertheless, that the long-term risk of dementia, including Alzheimer’s disease, had slightly decreased. They believed that patients with early-onset dementia or milder degrees of cognitive impairment would overlook, disregard, or misdiagnose milder symptoms of HZ, resulting in misclassification and an apparent non-causal decreased risk of dementia in those with HZ. They think the vast majority of patients received antiviral medications in this setting considering that their cohort was mostly identified based on antiviral prescriptions written at HZ-specific dosages. Since 1 multicenter observational cohort study,^[49]^ and 2 other East Asian studies^[43,44]^ particularly addressed the impact of antivirals for HZ, they discovered that any antiviral treatment was linked to a reduction in the relative risk of dementia of up to 45%. The recommended treatment may therefore serve to further minimize the risk of dementia. Moreover, Schmidt et al^[55]^ revealed that dementia risk was nearly doubled in the first year following cranial nerve HZ (HR 1.83; 95%CI = 1.03–3.25), with the ophthalmic nerve accounting for the majority of cases (83%; 990 of 1190). These findings are consistent with ours and those of Tsai et al.^[36]^

According to Schmidt et al,^[55]^ neuroinflammation and direct cerebral injury may act as a potential mediators in the association between HZ affecting the CNS and dementia. Even though vasculopathy may be implicated, integrating stroke diagnosis into the mediation model failed to lower HRs. Thus, patients with HZ who were diagnosed with stroke after the index date had higher HRs than those who had neither HZ nor stroke at baseline (HR 1.53; 95% CI = 1.09–2.15) within the first year. Hence, stroke was not a contributing factor. Even yet, they were unable to rule out the chance of a microinfarction, which is unrelated to clinical stroke. Acute or subacute effects of HZ, misrepresentation of reversible cognitive change brought on by the acute illness, or diagnostic bias resulting from intensive clinical examination in the acute phase may all be responsible for the particularly strong connection in the first year. The latter could explain the short-term elevation in dementia risk among stroke patients who also had HZ diagnoses from hospitals, especially ophthalmic HZ.

Some studies have discovered a correlation between gender and dementia, but since they merely focused on vascular dementia, their conclusions can indeed be relied on.^[53,56–58]^ Ruitenberg et al^[59]^ observed that the incidence of vascular dementia was higher for males than for women in all age ranges in a comprehensive population-based prospective cohort investigation. Contrarily, Schmidt et al,^[55]^ found the lowest HR in men (HR 0.89; 95% CI = 0.85–0.92 compared with 0.95; 95% CI = 0.92–0.97 in women).

Numerous studies have suggested that Amyloid-β peptide (Aβ) fibrilization and deposition as β-amyloid are hallmarks of AD pathology.^[21]^ These abnormal accumulations lead to the degeneration of synapses and neuronal brain cells (neurodegenerative dementia) and the impairment of cerebral blood flow (vascular dementia) as a result of atherosclerosis or vasculopathy. Amyloid formation is multifactorial and complex, involving interactions between environmental and host factors (age-associated changes such as immunosenescence, decreased glymphatic clearance, altered extracellular matrix proteins, and genetic variants); however, infectious agents have been proposed as triggers.^[21,60,61]^ The systemic inflammation brought on by viral infection causes brain reactions through microglial activation, aggravates the buildup of Ab and tau protein, and accelerates the development of dementia.^[62]^ Except for amylin accumulation, our results reported no significant correlation between HZV and the levels of the different dementia biomarkers we focused on in our study. Research by Buback et al^[23]^ published in 2020 explained the connection between amyloid, VZV, and dementia after investigating the synthesis of amyloid as well as intracellular amyloidogenic proteins between mock- and VZV-infected quiescent primary human spinal astrocytes (qHA-sps), concluding that VZV-infected qHA-sps produced intracellular amyloid and that the extracellular environment of these cells encouraged the formation of amyloid fibrils from cellular peptides, which may have been boosted by VZV gB peptides indicating that cells previously infected with VZV will continue to develop amyloidogenic peptides and amyloid persisted even after receiving acyclovir therapy, imply that VZV infection may contribute to the development of amyloid-associated diseases by increasing the toxic amyloid burden in conjunction with host and other environmental variables.^[23]^ Additionally, Bubak et al^[38]^ published in 2021, reported that zoster plasma includes factor(s) that, taken together, enhanced amyloid fibrillization. The zoster group exhibited considerably higher plasma levels of amyloid than healthy non-zoster controls, although there were no significant differences in the levels of Aβ40, Aβ42, Aβ42/Aβ40 ratios, or amylin. However, additional statistical studies showed that the amylin or Aβ42 levels in the zoster group were positive predictors of total amyloid levels. This shows that zoster plasma may contain a substance or factors that may enhance the amyloid fibrillization of Aβ42 or amylin. The association between Aβ42 and amyloid is positive, as is the correlation between amylin and amyloid. Alternately, or additionally, the zoster plasma may be deficient in one or more inhibitory factors that inhibit Aβ40 or amylin from producing amyloid.

In light of the previous assumptions, Lophatananon et al^[39]^ discovered a significant difference in the distribution of shingles between dementia incident cases and controls. They were able to obtain more thorough data for both dementia outcome and shingles exposure thanks to these data sources. After adjusting for age and sex, their findings revealed that there was a slight but non-significant increase in the risk of dementia in people with shingles diagnosed 3 years or more before dementia diagnosis. This was despite the fact that VZV has been proposed as a direct cause of dementia or that shingles may cause peripheral inflammation that may result in brain inflammation and the potential reactivation of Herpes Simplex Virus 1 (HSV1) or that VZV, like Cytomegalovirus (CMV), causes immune dysregulation, as proposed for the role of CMV in AD, by Stowe et al^[63]^ and Westman et al.^[64]^

Furthermore, Lopatko Lindman et al^[65]^ study found that the group with VZV infection had a somewhat higher risk of dementia than the group with HSV infection (HRs 1.61 and 1.38, respectively). In this substantially matched cohort trial, particular antiviral therapy directed against herpesviruses was related to an 11% lower incidence of dementia. However, compared to controls, receiving a herpes diagnosis without antiviral treatment was linked to a 50% increased risk of dementia. These findings are consistent with other register-based studies from Taiwan and South Korea that suggested that antiviral regimens may have a protective effect on dementia as well.^[43,44,66]^ To potentially lower the long-term risk of dementia, these findings may have possible clinical consequences, prompting doctors to be even more proactive in treating herpes reactivation symptoms with antiviral medications. Further classification of VZV infection into subtypes (herpes zoster ophthalmicus, and varicella) may be instructive when interpreting the data. Unfortunately, this subtype classification was not provided in this investigation. However, the control group in this study comprised both seropositive and seronegative status as the seropositivity status of the subjects is unknown, especially because > 95% of the population is projected to be VZV-positive and > 70% of the population is expected to carry HSV1.^[65]^ Because the controls have not been given a herpes diagnosis or have not undergone specialized medical treatment for this, it is plausible to presume that they are more immune resilient to herpes infections and have fewer bouts of symptomatic reactivations. Importantly, herpes diagnoses primarily reflect original infections with overt symptoms or symptomatic reactivation. Thus, the individuals with herpes diagnoses constitute a subgroup of those carrying the pathogen.

### 4.1. Strength and limitations

The overall quality was good in most of the studies included in our analysis. A good number of studies were subjected to analysis as 9 studies were included. Along with a decent sample size, as 3,090,813 patients were included in our analysis. Moreover, we found that some studies adjusted for some confounding factors for example, Warren et al,^[41]^ Tsai et al,^[36]^ Lopatko Lindman et al,^[65]^ and Choi et al.^[40]^

Our study shows some limitations. For, example all the studies included were non-randomized observational and hence might be subjected to bias. Prospective multicenter studies are needed to further evaluate the relationship between HZV and dementia development.

### 4.2. Future implications

It is not unexpected that there is no correlation between HZ and an increased risk of dementia, despite epidemiological evidence and biological mechanisms linking HZ to acute cerebrovascular episodes. However, we reported an increase in the prevalence of dementia among HZO groups (reactivation of latent infection). Thus, patients suffering from primary infections should be continuously assessed to prevent the development of HZO and consequently dementia. To further investigate associations, more multicenter randomized Studies are needed to support our findings. Since the middle of the millennium, research has been conducted on how herpes virus infection affects cognitive decline. In order to further investigate associations, future research should combine data from multiple large, prospectively collected datasets from different populations. It is important to minimize biases by, for instance, including laboratory-confirmed infection definitions, repeated regular assessments of the outcome, and thorough confounder control. A deeper comprehension of the intricate relationships between infections, immune system control, and the risk of chronic diseases may make it easier to identify vulnerable people and plan future interventions.

## 5. Conclusion

Our analysis of 9 studies with 3,090,813 patients revealed no statistically significant difference between the HZV group and no HZV group concerning the incidence of dementia, incidence of AD, and the increased level of Aβ40, Aβ42, and amyloid. However, we found a statistically significant association between HZV group and an increased level of Amylin compared with No HZV group. Also, no statistically significant difference was noticed when analyzing the incidence of HZV in the dementia group compared with no HZV group. On the other hand, our study found a statistically significant association between patients who have HZO and an increased incidence of dementia. Therefore, we concluded that HZO (reactivation of latent infection) increases the prevalence of dementia. To further investigate associations, more multicenter randomized Studies are needed to support our findings.

## Author contributions

**Conceptualization:** Rowan H. Elhalag, Karam R. Motawea, Marwan Abowafia.

**Data curation:** Rowan H. Elhalag, Karam R. Motawea, Nesreen Elsayed Talat, Samah S. Rouzan, Sarraa M. Reyad, Soliman M. Elsayed, Pensée Chébl, Jaffer Shah.

**Formal analysis:** Nesreen Elsayed Talat.

**Investigation:** Rowan H. Elhalag, Karam R. Motawea.

**Methodology:** Rowan H. Elhalag, Karam R. Motawea.

**Project administration:** Rowan H. Elhalag, Karam R. Motawea.

**Supervision:** Rowan H. Elhalag, Karam R. Motawea.

**Validation:** Karam R. Motawea.

**Visualization:** Karam R. Motawea, Soliman M. Elsayed, Jaffer Shah.

**Writing — original draft:** Rowan H. Elhalag, Nesreen Elsayed Talat, Samah S. Rouzan, Sarraa M. Reyad, Pensée Chébl.

**Writing — review & editing:** Rowan H. Elhalag, Karam R. Motawea, Marwan Abowafia.
